# Continuity of Caregivers’ Ensuring Medical Care and Care-Transition Preparedness: A Mediation Model

**DOI:** 10.1093/geroni/igab046.1073

**Published:** 2021-12-17

**Authors:** Nosaiba Rayan-Gharra, Orly Tonkikh, Nurit Gur-Yaish

**Affiliations:** 1 University of Haifa, Haifa, Hefa, Israel; 2 Oranim Academic College of Education, Haifa, HaZafon, Israel

## Abstract

Studies show that informal support provided during hospitalization is essential for communicating with the healthcare team and explaining medical care. Less is known about factors explaining family caregivers' Ensuring and Explaining Medical Care (EEMC) during hospitalization and its impact on care-transition-preparedness of patients in terms of their understanding of the explanations and instructions for continued care. This study examined whether EEMC during the current hospitalization mediates the association between involvement of the caregiver in ensuring and explaining medical care prior the current hospitalization and patients’ care-transition-preparedness for discharge. A prospective cohort study includes 456 internal-medicine-patients at a tertiary medical center in Israel, who were accompanied by an informal caregiver. Involvement in EEMC prior and during the hospitalization, covariates such as health literacy (HL) levels, demographic, health, and functional status were reported by the patients during the hospitalization; and care-transition-preparedness was reported by the patients in a week after discharge. After controlling for covariates, only high HL levels of patients and their caregivers were positively associated with EEMC during hospitalization and care-transition-preparedness (P<0.05). Moreover, mediation analysis indicated significant direct (B(unstandardized)=1.69; p=0.003) and indirect effect (Mediated effect (ME)=1.28; CI= 0.81 to 1.87) of prior involvement in EEMC on care-transition-preparedness through high EEMC during the current hospitalization, controlling for baseline characteristics of patients and their caregivers (total effect: B=2.95; p<0.001). These findings suggest that caregivers' experience and involvement prior the hospitalization may be an essential factor in improving EEMC during the current hospitalization, and in turn improve transition outcomes.

